# T1 and T2 mapping reveal contribution of hemorrhage in myocardial remodeling following acute myocardial infarction

**DOI:** 10.1186/1532-429X-18-S1-Q5

**Published:** 2016-01-27

**Authors:** Nilesh R Ghugre, Venkat Ramanan, Stephania Assimopoulos, Xiuling Qi, Jennifer Barry, Bradley H Strauss, Graham A Wright

**Affiliations:** 1 grid.17063.33Physical Sciences Platform, Sunnybrook Research Institute, Toronto, ON Canada; 2 grid.17063.33Department of Medical Biophysics, University of Toronto, Toronto, ON Canada; 3 grid.17063.33Biophysics and Physics, University of Toronto, Toronto, ON Canada; 4 grid.413104.30000000097431587Schulich Heart Research Program, Sunnybrook Health Sciences Centre, Toronto, ON Canada

## Background

Hemorrhage is a frequent complication in acute myocardial infarction (AMI) and is speculated to be an independent predictor of adverse outcomes [1]. In addition to infarct zone remodeling, the distal remote myocardium may experience alterations - vasodilator dysfunction, edema, and extracellular matrix (ECM) expansion [2-4]. The aim of our study was to understand the impact of hemorrhage on infarct as well as remote myocardial remodeling following an ischemic event. To this end, we employed quantitative T1 and T2 mapping to probe the underlying tissue alterations in a novel hemorrhagic model of AMI.

## Methods

Hemorrhage was artificially induced in a pig model by direct intracoronary injection of collagenase (col) [5]. Animals (N = 14) were divided into three groups and subjected to an LAD occlusion followed by reperfusion: Group 1 (N = 4) 45 min+saline (sal); Group 2 (N = 5): 8 min+col; and Group 3 (N = 5): 45 min+col. Imaging was serially performed on a 3T MRI scanner (MR 750, GE Healthcare) at baseline (healthy) and up to week 4 post-intervention. Pre- and post-contrast T1 values were quantified using a MOLLI sequence. Partition coefficient (λ) was estimated from the relation:(1/T1_myo,post_-1/T1_myo,pre_)/(1/T1_blood,post_-1/T1_blood,pre_). T2 mapping was performed using a T2-prepared spiral sequence. Hemorrhage was assessed from T2* maps obtained using a multi-echo gradient-echo acquisition. Infarcted and remote myocardial segments were evaluated based on LGE images.

## Results

**Figure 1:**
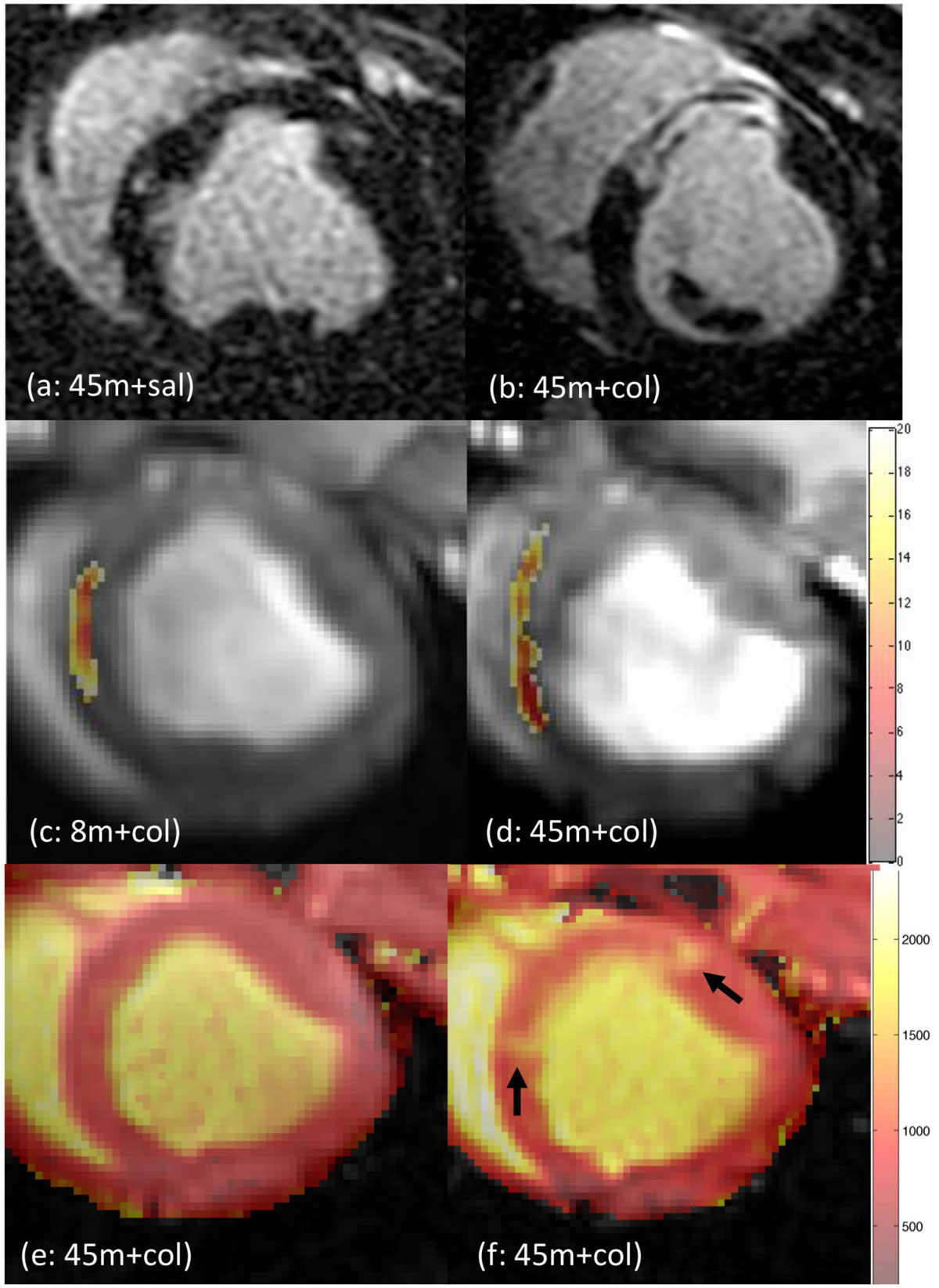
**Representative Images from the three groups**. (a) and (b) are LGE images from Groups 1 and 3, respectively, at day 1. (c) and (d) are T2*-weighted images with overlay of the hemorrhagic regions as identified by T2*<20 ms from Groups 2 and 3, respectively, at day 1 (colorbar indicates T2* values in ms.). (e) and (f) show pre contrast T1 maps in healthy state (baseline) and week 1, respectively, from Group 3 (arrows indicate edema).

**Figure 2:**
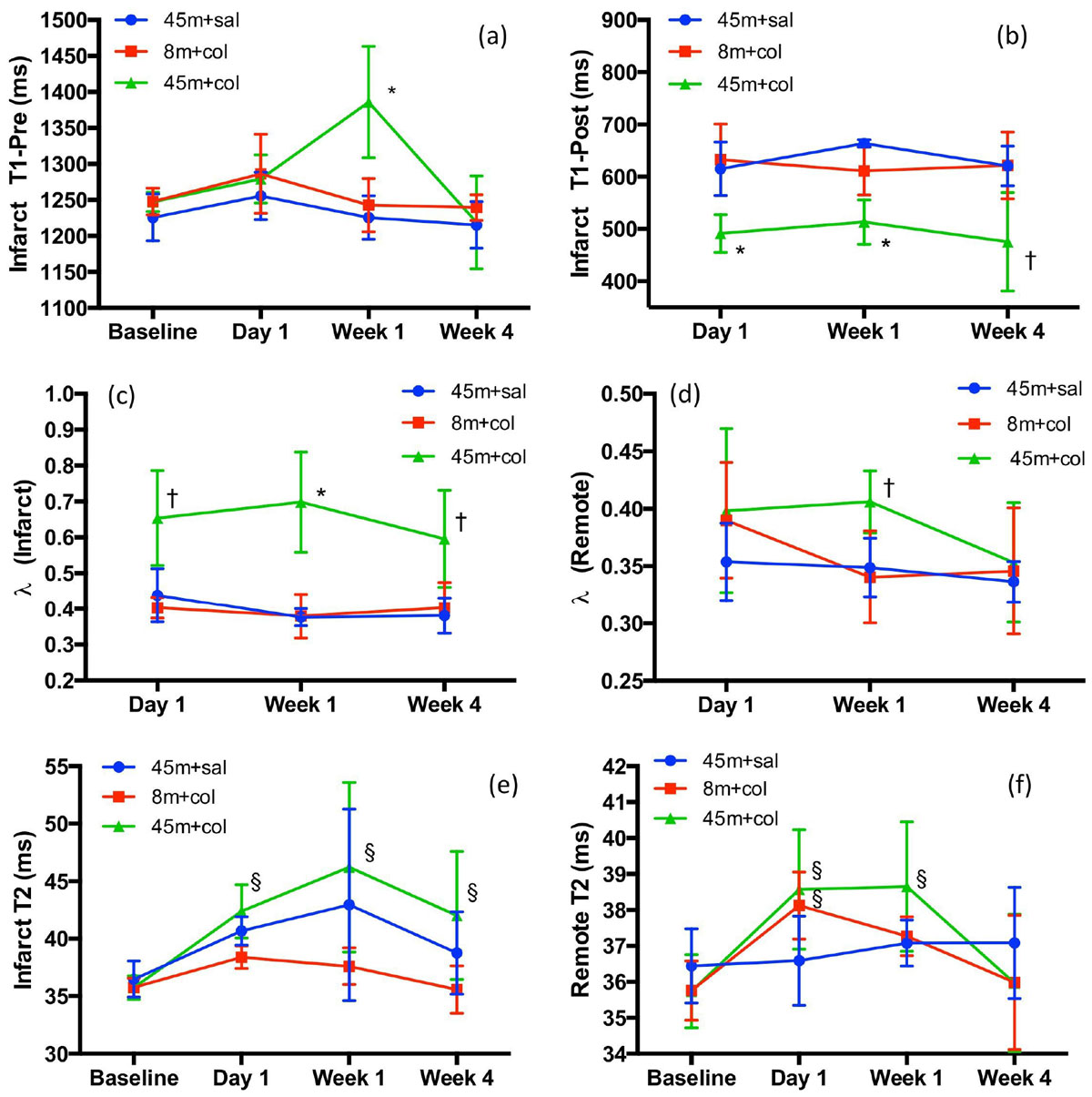
**Evolution of T1, partition coefficient (λ) and T2 in infarcted and remote myocardium in the three animal groups**. *p < 0.01; †p < 0.05 when Group 3 (45 m+col) was compared with the other two groups at a given time point; §p < 0.05 when compared with baseline from the same group.

LGE-based infarct size was significantly greater in group 3 compared to group 1 at all time points (p < 0.0001); group 2 was non-infarcted (Figure 1a,b). Low T2* values (<20 ms) confirmed the presence of hemorrhage in the collagenase groups 2 and 3 whereas group 1 was non-hemorrhagic (Figure 1c,d). At week 1, T1_pre_ in the infarct zone was significantly higher in group 3 compared to the other two groups, indicative of a greater edematous response (Figure 1e,f; Figure 2a). In group 3, T1_post_ in the infarct zone was significantly lower at all time points with respect to groups 1 and 2, suggesting extensive myocardial damage and fibrotic development; in agreement, infarct zone λ was also consistently higher in group 3 ((Figure 2b,c). Interestingly groups 2 and 3 demonstrated an elevation in remote zone λ at day 1 and week 1, potentially indicative of ECM alterations in the remote myocardium. Infarct T2 in group 3 was significantly elevated at all time points post-AMI (Figure 2e); T2 showed greater sensitivity to edema compared to T1_pre_ (30% vs 11% at week 1). Remote T2 fluctuations were similar to remote zone λ (Figure 2f); Remote T1_pre_ did not show any changes in time.

## Conclusions

Our study demonstrates that hemorrhage not only contributes to cellular and microvascular damage and inflammation but may further be responsible for edema and ECM expansion in distal remote myocardium. Early detection of remote tissue alterations will potentially aid better management of the high-risk patients who are prone to adverse long-term consequences.

